# Combination of tyrosine kinase inhibitors and the MCL1 inhibitor S63845 exerts synergistic antitumorigenic effects on CML cells

**DOI:** 10.1038/s41419-021-04154-0

**Published:** 2021-09-25

**Authors:** Alena Malyukova, Dorina Ujvari, Elham Yektaei-Karin, Ana Zovko, Harsha S. Madapura, Marton Keszei, Noemi Nagy, Kourosh Lotfi, Niclas Björn, Jonas Wallvik, Minori Tamai, Thao T. T. Nguyen, Koshi Akahane, Takeshi Inukai, Leif Stenke, Daniel Salamon

**Affiliations:** 1 Department of Medicine Solna, Karolinska Institute, Stockholm, Sweden; 2 Department of Women’s and Children’s Health, Karolinska Institute, Stockholm, Sweden; 3 Department of Microbiology, Tumor and Cell Biology, Karolinska Institute, Stockholm, Sweden; 4 Department of Cell and Molecular Biology, Karolinska Institute, Stockholm, Sweden; 5 Department of Hematology, Linköping University Hospital, Linköping, Sweden; 6 Department of Biomedical and Clinical Sciences, Linköping University, Linköping, Sweden; 7 Department of Public Health and Clinical Medicine, Umeå University, Umeå, Sweden; 8 Department of Pediatrics, School of Medicine, University of Yamanashi, Chuo, Japan; 9 Division of Hematology, Karolinska University Hospital Solna, Stockholm, Sweden

**Keywords:** Cancer stem cells, Targeted therapies, Preclinical research

## Abstract

Tyrosine kinase inhibitor (TKI) treatment has dramatically improved the survival of chronic myeloid leukemia (CML) patients, but measurable residual disease typically persists. To more effectively eradicate leukemia cells, simultaneous targeting of BCR-ABL1 and additional CML-related survival proteins has been proposed. Notably, several highly specific myeloid cell leukemia 1 (MCL1) inhibitors have recently entered clinical trials for various hematologic malignancies, although not for CML, reflecting the insensitivity of CML cell lines to single MCL1 inhibition. Here, we show that combining TKI (imatinib, nilotinib, dasatinib, or asciminib) treatment with the small-molecule MCL1 inhibitor S63845 exerted strong synergistic antiviability and proapoptotic effects on CML lines and CD34+ stem/progenitor cells isolated from untreated CML patients in chronic phase. Using wild-type BCR-ABL1-harboring CML lines and their T315I-mutated sublines (generated by CRISPR/Cas9-mediated homologous recombination), we prove that the synergistic proapoptotic effect of the drug combination depended on TKI-mediated BCR-ABL1 inhibition, but not on TKI-related off-target mechanisms. Moreover, we demonstrate that colony formation of CML but not normal hematopoietic stem/progenitor cells became markedly reduced upon combination treatment compared to imatinib monotherapy. Our results suggest that dual targeting of MCL1 and BCR-ABL1 activity may efficiently eradicate residual CML cells without affecting normal hematopoietic stem/progenitors.

## Introduction

Chronic myeloid leukemia (CML) is characterized by the presence of the BCR-ABL1 fusion oncoprotein, a constitutively active tyrosine kinase [1, 2]. Inhibition of BCR-ABL1 activity by imatinib or other specific tyrosine kinase inhibitors (TKIs) has revolutionized the management of CML, providing near to normal life expectancy for most patients [2, 3]. However, in spite of continuous TKI administration, a large majority of CML patients persistently display measurable residual disease and cannot stop their treatment without signs of relapse [4], reflecting the relative insensitivity of residual CML stem/progenitor cells to TKIs despite efficient inhibition of BCR-ABL1 activity [1, 2]. Several studies suggest that this BCR-ABL1-independent resistance is the consequence of the enhanced activity of various survival pathways in these cells. Therefore, the combination of BCR-ABL1 inhibition and drugs targeting additional key survival proteins of CML cells has been proposed [1, 2, 5].

Myeloid cell leukemia 1 (MCL1) is an important antiapoptotic member of the BCL2 gene family [6] that is frequently overexpressed in several human cancer types, including CML [7–9]. More importantly, downregulation of MCL1 expression with an antisense oligonucleotide induced apoptosis in the K562 CML cell line [9], suggesting that MCL1 might be one of the BCR-ABL1-independent survival proteins in CML stem/progenitor cells.

Several highly potent and selective small-molecule MCL1 inhibitors have recently been discovered and advanced to clinical trials [7, 8]. One of these novel inhibitors, S63845, is effective in the low nanomolar range as a single agent or in combination with other anticancer drugs on most myeloma, lymphoma and acute myeloid leukemia derived cell lines in vitro and in vivo, without affecting normal hematopoietic progenitor cells [10–12]. It is tolerated in mice at efficacious doses [10] and its derivative MIK665/S64315 has already entered clinical trials in these hematological malignancies and in myelodysplastic syndrome [7, 8]. In contrast, CML cell lines were relatively insensitive to single S63845 treatment, a finding suggested to be linked to their consistently high BCL-xL levels [10]. Notably, in the absence of cytokines, TKI treatment was shown to downregulate the expression of BCL-xL [13, 14] as well as several other antiapoptotic members of the BCL2 and baculoviral IAP repeat containing (BIRC) gene families in CML cells [14] leading us to analyze whether the addition of a small-molecule MCL1 inhibitor such as S63845 might enhances the antitumorigenic effects of TKIs on CML cells.

## Materials and methods

### Reagents

Imatinib (17 mM stock; Sigma-Aldrich) was dissolved in water. Asciminib, nilotinib (20 mM and 10 mM stocks, respectively; MedChemExpress), dasatinib, S63845 (10 mM stocks; Selleckchem), and Z-VAD-FMK (20 mM stock; Calbiochem) were dissolved in dimethyl sulfoxide (DMSO). DMSO concentration was equalized in each well of a particular experiment.

### Isolation of primary human CD34+ CML and normal hematopoietic stem/progenitor cells

Peripheral blood mononuclear cells (PBMCs) were separated by leukapheresis from the peripheral blood of untreated CML patients at diagnosis in the chronic phase of the disease. CD34+ cells were isolated from PBMCs using the Dead Cell Removal Kit and the human CD34 MicroBead Kit (both from Miltenyi Biotec), according to the manufacturer’s instructions.

Primary human normal bone marrow CD34+ hematopoietic stem/progenitor cells were obtained from American Type Culture Collection (Cat. No.: PCS-800-012) or from Lonza (Cat. No.: 2M-101), and kept in culture for 48 h in StemSpan SFEM II Medium supplemented with StemSpan CD34+ Expansion Supplement and 175 nM UM171 (all from STEMCELL Technologies) before starting the colony forming assay. Viability of normal bone marrow CD34+ hematopoietic stem/progenitor cells at the start of the colony forming assay was always above 95%.

Flow cytometric analysis using FITC-conjugated mouse anti-human-CD34 (clone AC136; Miltenyi Biotec; Cat. No.: 130-081-001) or FITC-conjugated mouse IgG2a (isotype control; Miltenyi Biotec; Cat. No.: 130-091-837) antibodies confirmed that in all cases more than 95% of the isolated cells were CD34+.

### Cell culture and treatment

The JURL-MK1 [15], K562 [16], and KCL-22 [17] CML lines were obtained from the German Collection of Microorganisms and Cell Cultures (DSMZ). TCCS is a CML cell line missing the normal ABL1 gene [18]. Imatinib resistant K562 and TCCS sublines harboring the T315I BCR-ABL1 mutation (K562^T315I^ and TCCS^T315I^) were generated by homologous recombination (HR) using the CRISPR/Cas9 system [19]. Characterization of the parental and T315I-mutated TCCS sublines has been previously published [19], while the sequence analysis of the genomic region harboring codon 315 of the ABL1 gene of the parental and T315I-mutated K562 sublines used in this study are presented in Supplementary Fig. 1. All cell lines were cultured in RPMI 1640 medium containing 10% heat inactivated fetal bovine serum and 1 mM L-glutamine (complete medium). All cell lines were PCR tested and found to be mycoplasma free.

**Fig. 1:**
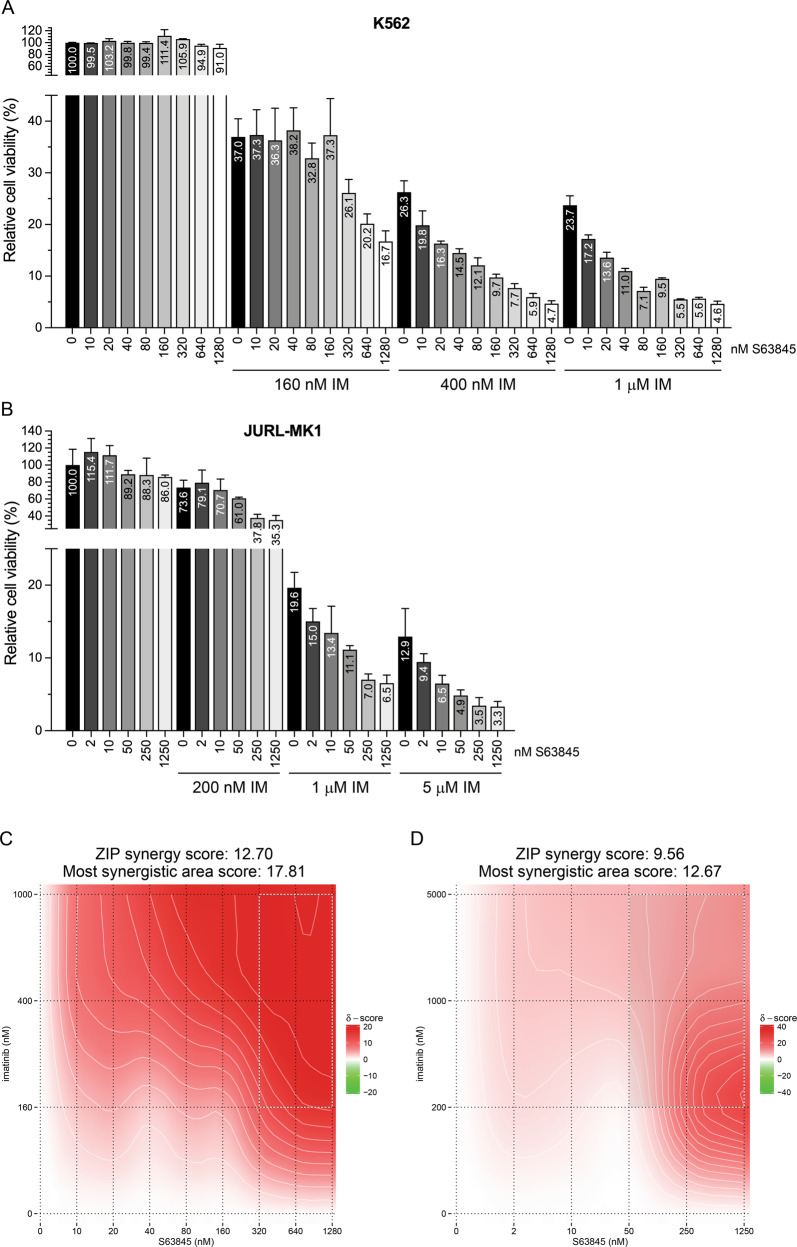
Effects of S63845 and imatinib administered as single drug or in combination on the viability of K562 and JURL-MK1 cells. Relative viability (**A**, **B**) and synergy maps (**C**, **D**) of K562 (**A**, **C**) and JURL-MK1 (**B**, **D**) cells left untreated or treated for 48 h (**A**, **C**) or 32 h (**B**, **D**) with the indicated concentrations of imatinib (IM) and/or S63845, analyzed by alamarBlue (**A**, **C**) or SYTOX Green (**B**, **D**) assays. Data represent mean with standard deviation derived from three technical replicates. Experiments were performed at least twice with similar results.

K562 cells seeded at 2 × 10^4^ cells/well in triplicates, in 96-well plates in complete medium, were left untreated or treated as indicated, followed by the quantification of relative cell viability using AlamarBlue Assay (Thermo Fisher Scientific) according to the manufacturer’s instructions.

JURL-MK1 cells, parental and T315I-mutated TCCS cells seeded at 3 × 10^4^ cells/well, parental and T315I-mutated K562 cells seeded at 2 × 10^4^ cells/well, and KCL-22 cells seeded at 4 × 10^4^ cells/well in triplicates, in 96-well plates, in phenol-red free complete medium supplemented with 150 nM SYTOX Green Nucleic Acid Stain (Thermo Fisher Scientific) or 250 nM IncuCyte Cytotox Green Dye, or 2.5 μM IncuCyte Caspase-3/7 Green Reagent for Apoptosis (both from Essen Bioscience), were left untreated or treated as indicated, followed by the quantification of the number of SYTOX Green negative (viable) cells, or the ratio of Cytotox Green positive (dead) or active caspase-3/7 positive (apoptotic) cells, with fluorescence live cell microscopy. If needed, K562 cells were pretreated for 1 h with 40 μM Z-VAD-FMK.

Primary human CD34+ CML stem/progenitor cells seeded in triplicates in StemSpan SFEM Medium (STEMCELL Technologies) (without supplementation or supplemented with 0.5 ng/ml each of recombinant human stem cell factor (SCF), Flt3-ligand (FL), and thrombopoietin (TPO) (all from Peprotech)) at 3 × 10^4^ cells/well, in 96-well plates were left untreated or treated as indicated, followed by the quantification of the number of viable cells under microscope, performed blind by two independent observers using a hemocytometer and trypan blue exclusion.

Primary human CD34+ CML stem/progenitor cells seeded in StemSpan SFEM medium supplemented with 0.5 ng/ml each of SCF, FL and TPO in addition to either 250 nM SYTOX Green Nucleic Acid Stain (in duplicate) or 2.5 μM IncuCyte Caspase-3/7 Green Reagent for Apoptosis (in triplicate) at 3 × 10^4^ cells/well, in 96-well plates were left untreated or treated as indicated, followed by the quantification of the number of SYTOX Green negative, or the ratio of active caspase-3/7 positive cells, using fluorescence live cell microscopy.

K562 and TCCS cells seeded at 9 × 10^5^ cells/well in complete medium, in six-well plates, or primary human CD34+ CML stem/progenitor cells seeded in StemSpan SFEM Medium supplemented with 0.5 ng/ml each of SCF, FL and TPO, at 2.5 × 10^5^ cells/well, in 24-well plates, were left untreated or treated as indicated, followed by the analysis of total cell lysates with immunoblotting.

### Immunoblotting

Total cell lysates were analyzed by SDS-polyacrylamide gel electrophoresis and immunoblotting, with the antibodies listed in Supplementary Table 1.

### Fluorescence live cell microscopy

Fluorescence live cell microscopy was performed with an IncuCyte S3 Live Cell Analysis System (Essen Bioscience). Nine planes of view were collected per well, using the 20× objective. The obtained data were analyzed with the IncuCyte S3 Cell-by-Cell Analysis Software Module (Essen Bioscience).

### Colony forming assay

CML or normal CD34+ hematopoietic stem/progenitor cells were seeded in triplicates in 24-well plates in MethoCult H4435 Enriched Medium (containing SCF, interleukin 3, interleukin 6, erythropoietin, granulocyte colony-stimulating factor and granulocyte-macrophage colony-stimulating factor (Stemcell Technologies)) in the absence or presence of the indicated concentrations of imatinib and/or S63845. Whole wells were photographed after 12–14 days using an IncuCyte S3 Live Cell Analysis System (Essen Bioscience). Colonies were analyzed and quantified using ImageJ (ImageJ, National Institutes of Health, Bethesda, MD, http://rsb.info.nih.gov/ij/) with customized macros. Minimum colony size, intensity threshold and other parameters were optimized based on preliminary manual counts.

### Synergy calculations

Calculation of the expected drug combination responses (based on the ZIP reference model) and synergy scoring were carried out using SynergyFinder version 2.0 [20].

### Statistical analysis

Statistical analysis was performed with GraphPad Prism 8.0. All data were log-transformed, and normal distribution was challenged with the Kolmogorov–Smirnov test. According to the distribution of the data, two-tailed paired *T*-test or Wilcoxon signed-rank test was used to determine if there was a statistically significant difference between the treatment groups. *P* < 0.05 was considered as significant.

## Results

### Combination of small-molecule MCL1 inhibition and TKI treatment synergistically induces concurrent strong apoptotic and variable levels of GSDME-dependent pyroptotic cell death in CML cell lines

To analyze the potential interaction between imatinib and S63845 in CML cells, we first tested the effects of different concentrations of the two drugs and their combinations on the viability of the well-characterized, imatinib-sensitive CML lines K562 and JURL-MK1, using the alamarBlue and SYTOX Green assays, respectively (Fig. 1). In line with previous observations [10], imatinib treatment alone markedly reduced the viability of both cell lines, while S63845 as a single drug did not exert any significant effect on K562 cells, and only minimally reduced the viability of JURL-MK1 cells. In contrast, combined treatment with pharmacologically relevant concentrations of imatinib and down to two-digit nanomolar concentrations of S63845 reduced the viability of both cell lines markedly stronger than imatinib monotherapy, fulfilling the criteria of a strong synergistic interaction between the two drugs (Fig. 1C, D).

**Fig. 2:**
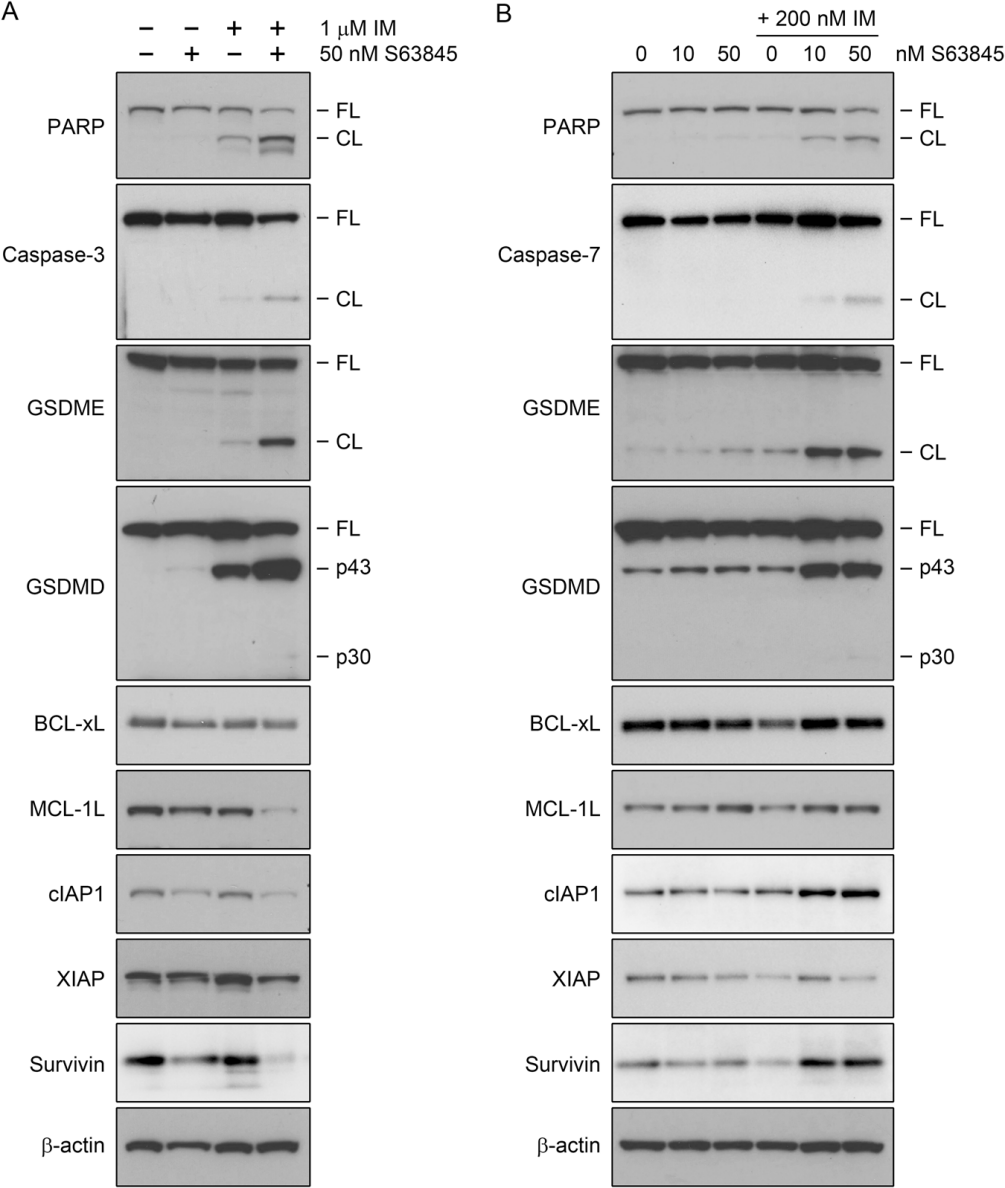
Effects of S63845 and imatinib administered as single drug or in combination on the expression of apoptotic and pyroptotic markers and key antiapoptotic members of the BCL2 and BIRC gene families in K562 and TCCS cells. Immunoblot analysis of PARP, caspase-3, caspase-7, GSDME, GSDMD, BCL-xL, MCL-1L (the longest isoform of MCL1), cIAP1, XIAP, survivin and β-actin protein expression in total cell extracts of K562 (**A**) and TCCS (**B**) cells left untreated (−) or treated (+) for 24 h (**A**) or 16 h (**B**) with the indicated concentrations of imatinib (IM) and/or S63845. Cleaved caspase-3 could not be detected in any lysates of TCCS, while cleaved caspase-7 was not detectable in any lysates of K562 cells (not shown). BCL2 and cIAP2 could not be detected in any lysates of either of the cell lines (not shown). Experiments were performed at least twice with similar results. Representative blots are shown. FL full length, CL cleaved (89 kDa C-terminal cleavage product of PARP or large subunits of cleaved caspase-3/7 or N-terminal cleavage product of GSDME), p43 p43 GSDMD fragment, p30 p30 GSDMD fragment.

To study whether S63845 might exert its antiviability effect through the enhancement of imatinib-induced apoptosis of CML cells, we tested the impact of the two drugs, alone or in combination on caspase-3/7 activation (cleavage) and caspase-specific cleavage of poly(ADP-ribose) polymerase (PARP) [21–23] in K562 and TCCS cells by western blot (Fig. 2). PARP cleavage or caspase-3/7 activation could not be detected in either of the cell lines left untreated or treated only with S63845. Imatinib monotherapy only minimally induced the cleavages of PARP and caspase-3 (but not caspase-7) in K562, while it did not exert any effect in TCCS cells. On the other hand, combination treatment moderately or strongly induced the activation of caspase-3 (but not caspase-7) in K562, and caspase-7 (but not caspase-3) in TCCS cells, and the cleavage of PARP in both cell lines.

To reveal any potential correlation between the induction of apoptotic markers and expression of key antiapoptotic factors, we measured the expression of some of the most important antiapoptotic members of the BCL2 (BCL2, BCL-xL and MCL1) and BIRC (cIAP1, cIAP2, XIAP and survivin) gene families in the previously analyzed cell lysates of K562 and TCCS cells (Fig. 2). Interestingly, the western blot analysis revealed diverse expression changes in the two cell lines. For example, combination treatment strongly downregulated MCL1, cIAP1 and survivin expression in K562, while minimally or moderately upregulated in TCCS cells. Furthermore, imatinib monotherapy did not affect the expression of any of the antiapoptotic proteins in K562 cells, whereas it minimally or moderately downregulated BCL-xL, XIAP and survivin expression in TCCS cells. Interestingly, BCL2 and cIAP2 proteins could not be detected in either of the cell lines (data not shown).

**Fig. 3:**
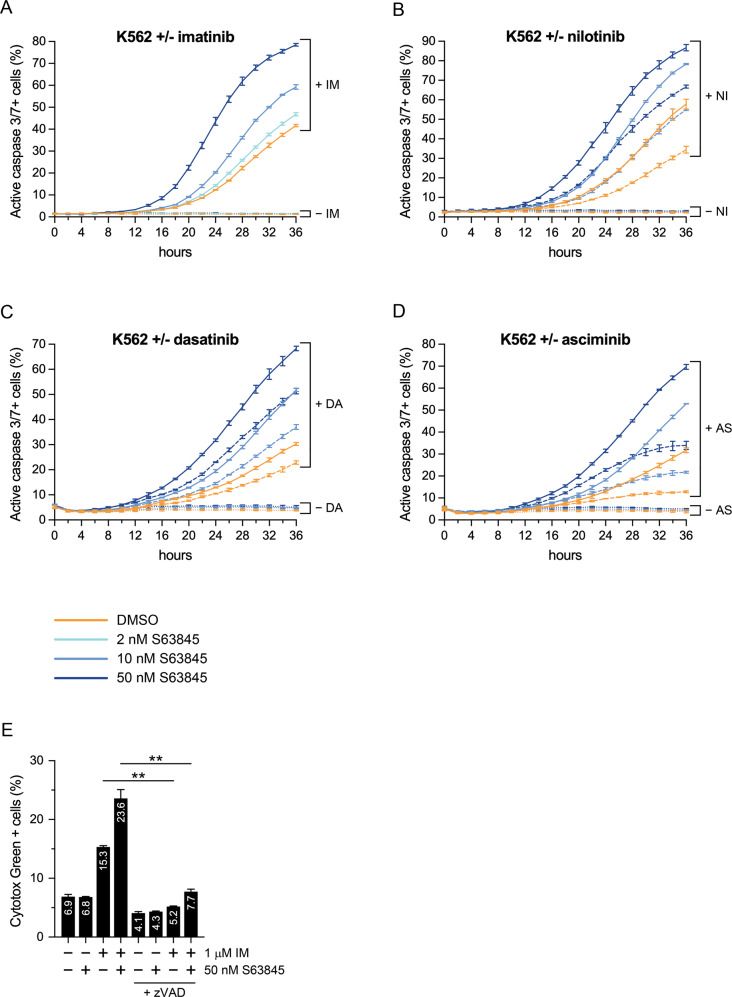
Combination treatment with S63845 and a TKI synergistically induces caspase-3/7 activation and caspase-dependent death in K562 cells. Proportion of active caspase-3/7 positive K562 cells left untreated or treated for the indicated hours with the indicated concentrations of S63845, in the absence (dotted lines) or presence of 1 μM (solid lines) imatinib (IM) (**A**), 20 nM (dashed lines) or 100 nM (solid lines) nilotinib (NI) (**B**), 1 nM (dashed lines) or 5 nM (solid lines) dasatinib (DA) (**C**), or 4 nM (dashed lines) or 20 nM (solid lines) asciminib (AS) (**D**) analyzed by fluorescence live cell microscopy. Data represent mean with standard deviation derived from three technical replicates. Experiments were performed at least twice with similar results. **E** Proportion of Cytotox Green positive K562 cells left untreated (−) or treated (+) for 36 h with 1 μM imatinib (IM) and/or 50 nM S63845, in the absence (−) or presence (+) of 40 μM Z-VAD-FMK (zVAD), analyzed by fluorescence live cell microscopy. Data represent mean with standard deviation derived from three independent experiments. **P < 0.01.

Next, we analyzed the effects of the drugs on the ratio of activated caspase-3/7 positive cells (as a marker of apoptosis) in the K562 (Fig. 3A) and KCL-22 (Supplementary Fig. 2) CML lines with fluorescence live cell microscopy. Again, imatinib alone only moderately increased apoptotic rates, while S63845 monotherapy induced none (K562) or minimal (KCL-22) level of apoptosis in the cell lines. On the other hand, combination treatment with pharmacologically relevant concentration of imatinib and low nanomolar concentrations of S63845 induced strong proapoptotic response with a rapid (K562) or a late onset (KCL-22) kinetics in both of the CML lines. Similarly to imatinib, the proapoptotic effect of two second-generation ATP-competitive TKIs nilotinib and dasatinib, and the highly selective, allosteric ABL1 kinase inhibitor asciminib [24] were also markedly enhanced by S63845 treatment, compared to TKI monotherapy on K562 cells (Fig. 3B–D). Since asciminib does not inhibit the potential secondary targets of ATP-competitive TKIs, our results suggest that the synergistic effect of the drug combinations is linked to ABL1 kinase inhibition, rather than due to the off-target effects of the TKIs [25, 26].

To analyze the functional relevance of caspase activation, the effect of imatinib and S63845 on the rate of K562 cell death was analyzed both in the absence or presence of the pan-caspase inhibitor Z-VAD-FMK (zVAD) [27] (Fig. 3E). In the absence of zVAD, S63845 monotherapy did not exert any effect, while imatinib monotherapy moderately upregulated and the drug combination synergistically further increased cell death rate. These cytotoxic effects were significantly blocked by the presence of zVAD, proving that the cell death induced by imatinib monotherapy or the combination treatment is mainly caspase-dependent.

**Fig. 4:**
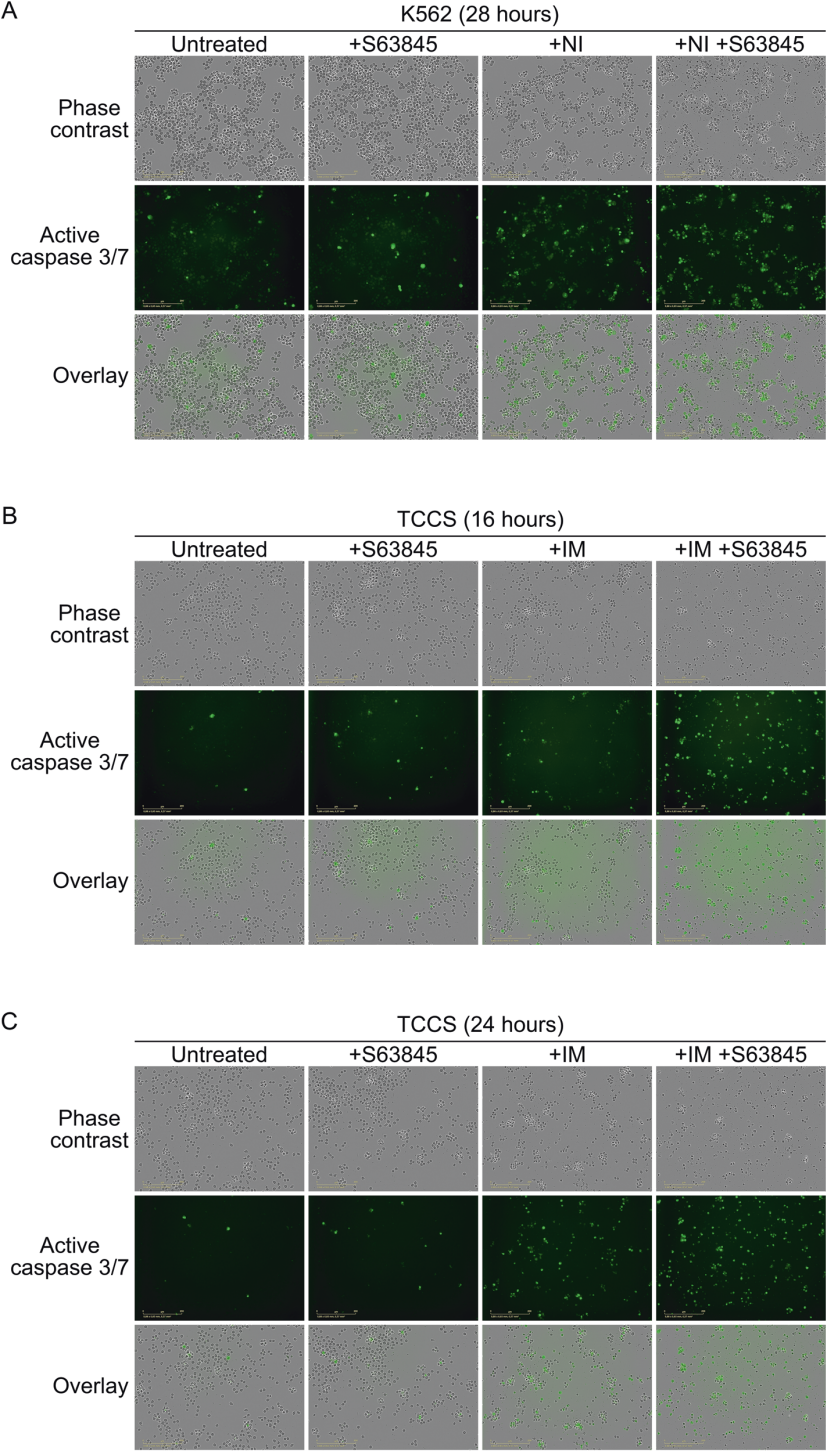
Effects of S63845 and TKIs administered as single drug or in combination on the morphology of K562 and TCCS cells. Representative fluorescence live cell microscopy images of K562 (**A**) and TCCS (**B**, **C**) cells left untreated or treated with 50 nM S63845 and/or 100 nM nilotinib (NI) for 24 h (**A**) or 50 nM S63845 and/or 200 nM imatinib (IM) for 16 h (**B**) or 24 h (**C**) in the presence of IncuCyte Caspase-3/7 Green Dye for Apoptosis. Experiments were performed at least twice with similar results.

Although, the majority of cell death induced by the drug combination showed the characteristic morphology of apoptosis [28], we also observed several active caspase-3/7 positive cells with cytoplasmic swelling, distinct from apoptotic blebbing in TCCS, and in much lower frequency in K562 cells (Fig. 4). Indeed, western blotting showed that while S63845 monotherapy did not induce (or only minimally upregulated) the cleavages of gasdermin E (GSDME) and gasdermin D (GSDMD) in the two cell lines, and imatinib monotherapy only minimally induced the caspase-3-specific cleavage of GSDME [29] in both lines, and moderately induced the production of the p43 GSDMD fragment (generated by the cleavage of GSDMD by activated caspase-3/7) [30] in K562 but not in TCCS cells, combination treatment markedly induced GSDME cleavage and the production of the p43 GSDMD fragment in both cell lines (Fig. 2). Furthermore, none of the treatments induced the production of the p30 GSDMD fragment (generated by the cleavage of GSDMD by inflammatory caspases) [31] in either of the cell lines (Fig. 2). Since GSDME cleavage by caspase-3 mediates both pyroptosis and apoptosis [32], while the caspase-3/7-specific cleavage inactivates GSDMD and therefore inhibits pyroptosis [30], our results suggest that combination treatment induces concurrent strong apoptotic and variable levels of GSDME-dependent pyroptotic cell death in CML cell lines.

### The synergistic proapoptotic interaction of MCL1 inhibition and TKI treatment in CML cells depends on TKI-mediated BCR-ABL1 inhibition and is not generated by TKI-related off-target mechanisms

**Fig. 5:**
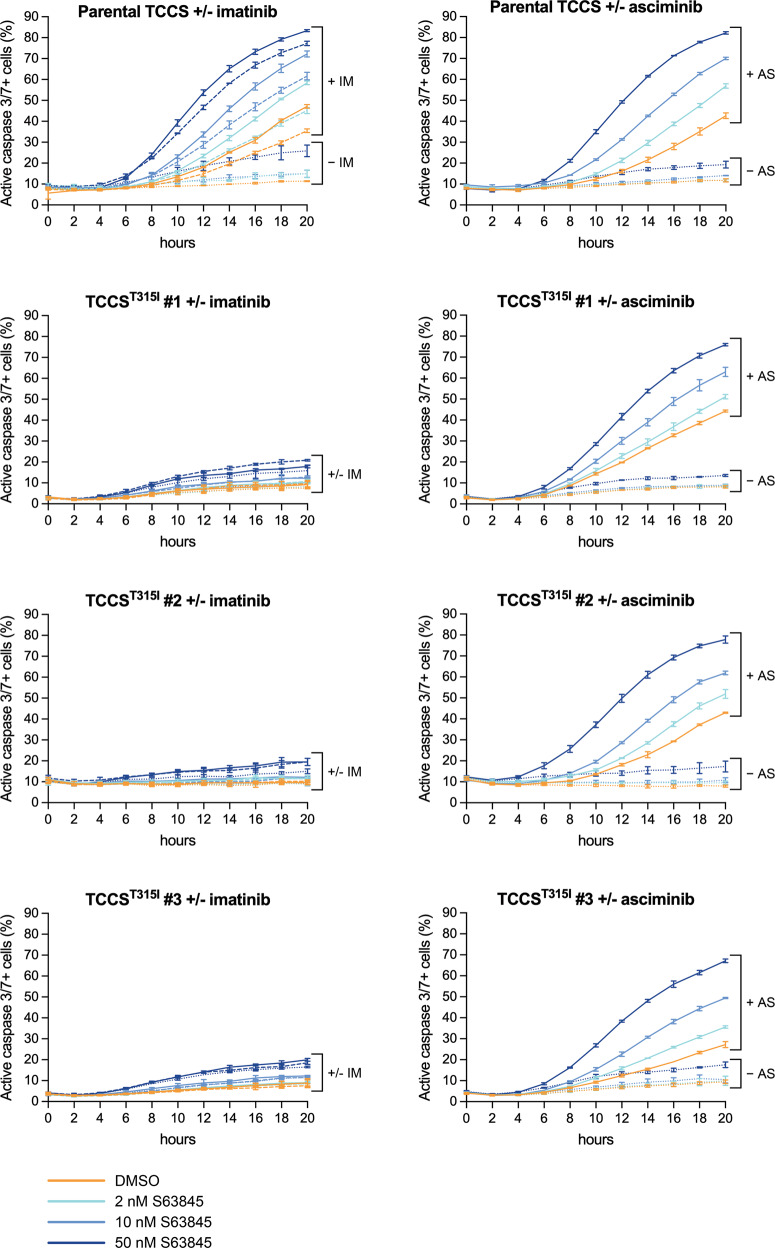
Effects of S63845 and TKIs administered as single drug or in combination on the apoptotic rate of the parental TCCS line and its imatinib resistant sublines harboring the T315I mutation in BCR-ABL1. Proportion of active caspase-3/7 positive cells in the parental TCCS line and its T315I mutation harboring sublines left untreated or treated for the indicated hours with the indicated concentrations of S63845 in the absence (dotted lines) or presence of 200 nM (dashed lines) or 1 μM (solid lines) imatinib (IM) (left panels), or in the absence (dotted lines) or presence of 50 nM (solid lines) asciminib (AS) (right panels), analyzed by fluorescence live cell microscopy. Data represent mean with standard deviation derived from three technical replicates. Experiments were performed at least twice with similar results.

**Fig. 6:**
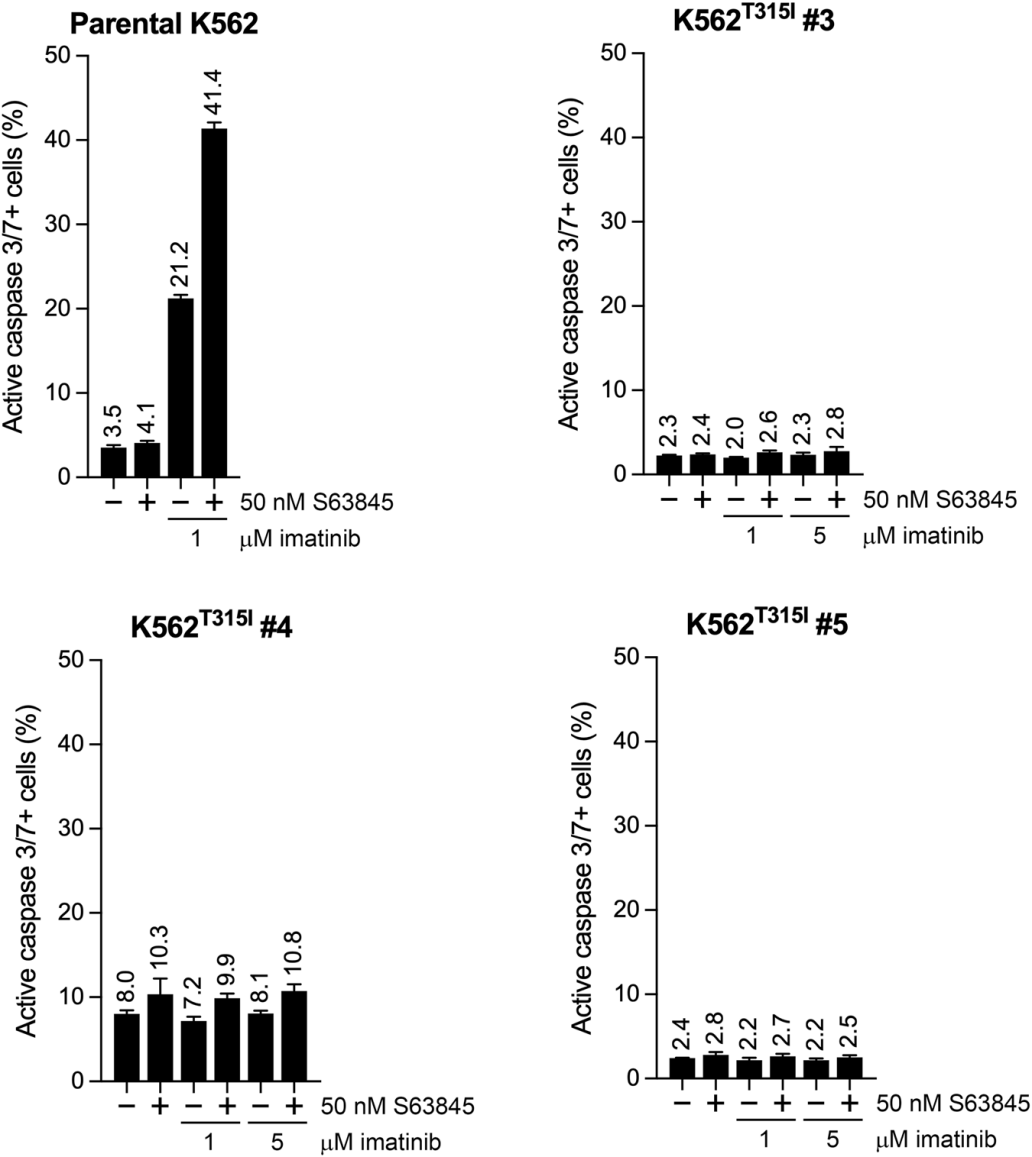
Effects of S63845 and imatinib administered as single drug or in combination on the apoptotic rate of the parental K562 line and its imatinib resistant sublines harboring the T315I mutation in BCR-ABL1. Proportion of active caspase-3/7 positive cells in the parental K562 line and its T315I mutation harboring sublines left untreated or treated for 60 h with 50 nM S63845 in the absence or presence of the indicated concentrations of imatinib, analyzed by fluorescence live cell microscopy. Data represent mean with standard deviation derived from three technical replicates. Experiments were performed at least twice with similar results.

To clearly prove that the potential off-target effects of imatinib [25, 26] do not play a key role in the synergistic interaction of MCL1 inhibition and imatinib treatment, we analyzed the effects of the two drugs and their combinations on the imatinib-sensitive parental TCCS and K562 CML lines (harboring wild-type BCR-ABL1) and their imatinib resistant sublines harboring the T315I mutation in BCR-ABL1 (TCCS^T315I^ and K562^T315I^), generated by HR using the CRISPR/Cas9 system [19]. In line with our previous results, the combination of imatinib and S63845 induced strong synergistic proapoptotic responses in the two parental cell lines, while both monotherapies and the combination treatment only minimally affected the apoptotic rate of the imatinib resistant sublines (Figs. 5 and 6). In contrast, combining low nanomolar concentrations of S63845 with 50 nM asciminib exerted a nearly equally strong synergistic proapoptotic effect both on the parental and the mutant TCCS sublines (Fig. 5), reflecting the moderately decreased but still retained inhibitory activity of asciminib on the T315I mutation. These results prove that off-target effects of imatinib do not play a key role, while inhibition of BCR-ABL1 signaling by TKIs is essential for the strong synergistic effect of the drug combination in CML cells.

### Small-molecule MCL1 inhibition strongly and rapidly enhances the antiviability effect of imatinib on primary human CD34+ CML stem/progenitor cells

**Fig. 7:**
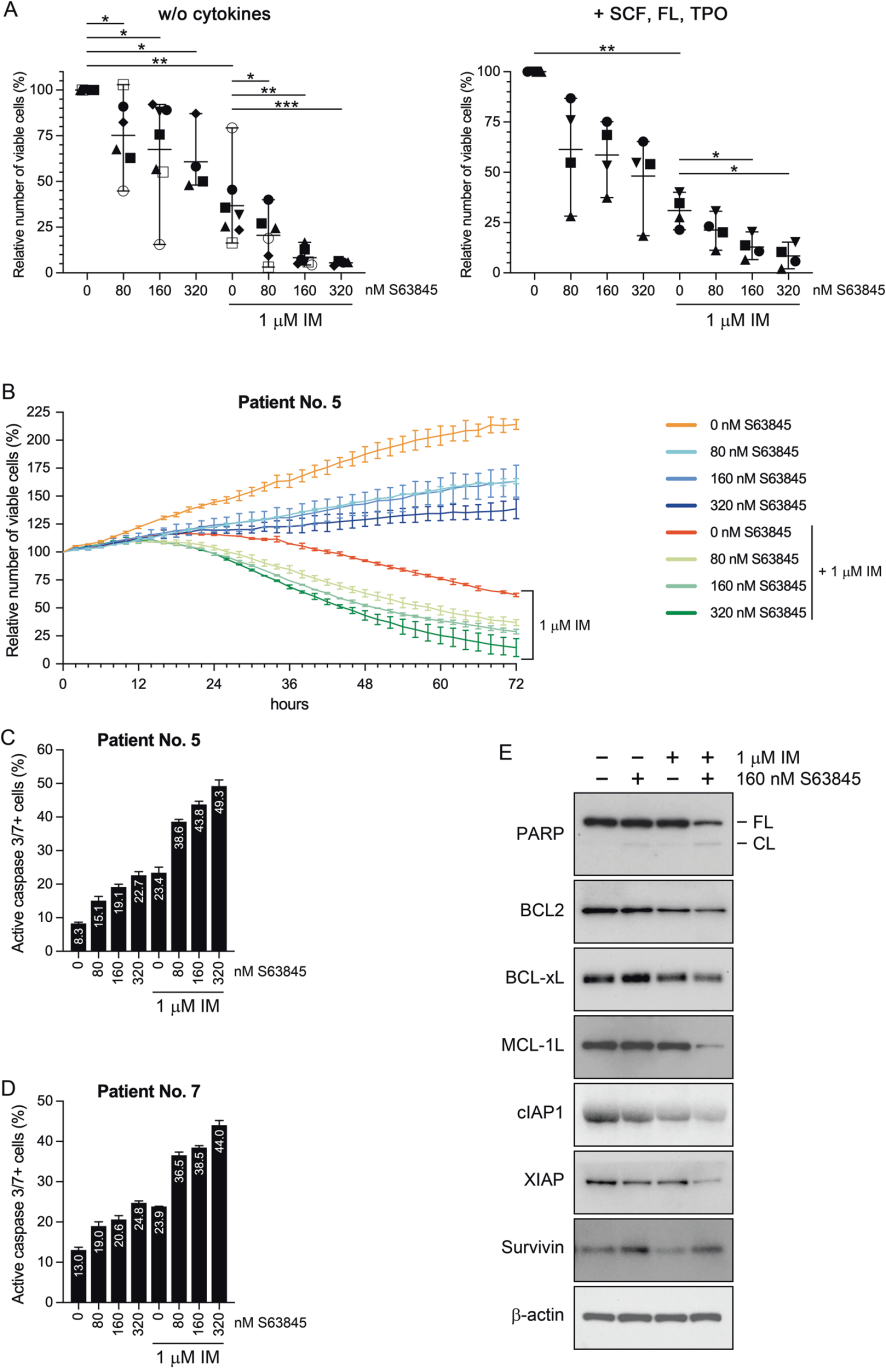
Effects of S63845 and imatinib administered as single drug or in combination on the viability, apoptotic rate and antiapoptotic protein expression of primary human CD34+ stem/progenitor cells obtained from the peripheral blood of untreated CML patients in chronic phase. **A** Relative numbers of viable CD34+ stem/progenitor cells obtained from the peripheral blood of untreated CML patients in chronic phase and left untreated or treated ex vivo for 96 h with 1 μM imatinib (IM) and/or the indicated concentrations of S63845 in unsupplemented (left panel; *n* = 7 for 0 and 160 nM, *n* = 6 for 80 nM, and *n* = 4 for 320 nM S63845) or SCF, FL and TPO (0.5 ng/ml each) supplemented (right panel; *n* = 4 for all S63845 concentrations) StemSpan SFEM Medium. Data represent mean with range derived from four to seven independent experiments. *P < 0.05; **P < 0.01; ***P < 0.001. Patient no. 1: ⚫; patient no. 2: ◼; patient no. 3: ▲; patient no. 4: ▼; patient no. 5: ◆; patient no. 6: ○; patient no. 7: ⬜. **B** Relative numbers of viable (SYTOX Green negative) CD34+ stem/progenitor cells obtained from the peripheral blood of CML patient No. 5 and left untreated or treated ex vivo for the indicated hours with 1 μM imatinib (IM) and/or the indicated concentrations of S63845 (represented by different colors) in StemSpan SFEM Medium supplemented with SCF, FL and TPO (0.5 ng/ml each), analyzed by fluorescence live cell microscopy. Proportion of active caspase-3/7 positive CD34+ stem/progenitor cells obtained from the peripheral blood of CML patient Nos. 5 (**C**) and 7 (**D**), and left untreated or treated ex vivo for 24 h with 1 μM imatinib (IM) and/or the indicated concentrations of S63845 in StemSpan SFEM Medium supplemented with SCF, FL and TPO (0.5 ng/ml each), analyzed by fluorescence live cell microscopy. Data shown in **B**–**D** represent mean with standard deviation derived from two (**B**) or three (**C**, **D**) technical replicates. **E** Immunoblot analysis of PARP, BCL2, BCL-xL, MCL-1L, cIAP1, XIAP, survivin and β-actin protein expression in total cell extracts of CD34+ stem/progenitor cells obtained from the peripheral blood of CML patient No. 2, and left untreated or treated ex vivo for 24 h with 1 μM imatinib (IM) and/or 160 nM S63845 in StemSpan SFEM Medium supplemented with SCF, FL and TPO (0.5 ng/ml each). cIAP2, the p30 GSDMD fragment, and the full length and cleaved GSDME proteins were not detectable in any of the lysates (not shown). FL full length, CL 89 kDa C-terminal cleavage product of PARP.

Next, we analyzed if S63845 treatment might also affect the viability of CML stem/progenitor cells in the absence or presence of imatinib. Therefore, we assessed the viability of primary human CD34+ CML stem/progenitor cells isolated from the peripheral blood of seven untreated CML patients in chronic phase of the disease and treated for 96 h with either of the drugs or their combination in ex vivo liquid cultures in the absence (Fig. 7A and Supplementary Fig. 3A, left panels) or presence (Fig. 7A and Supplementary Fig. 3A, right panels) of the early acting cytokines SCF, FL and TPO [33]. Treatment with 1 μM imatinib as a single agent reduced the number of viable cells in all assessed samples by a mean of 63% in the absence and 69% in the presence of cytokines. S63845 monotherapy also exerted a similar, but less pronounced dose-dependent inhibitory effect on viable cell numbers. However, in all the samples tested, both in the absence or presence of cytokines, combined treatment with 1 μM imatinib and two- to low three-digit nanomolar S63845 reduced the number of viable CML stem/progenitor cells markedly stronger (with up to 95% mean inhibition) than either of the monotherapies. Fluorescence live cell imaging analysis confirmed these results and also showed that the drug combination rapidly exerted its inhibitory effect on CML stem/progenitor cells (Fig. 7B).

### The antiviability effect of S63845 is exerted through the induction of apoptosis in primary human CD34+ CML stem/progenitor cells

To analyze if S63845 treatment might induce apoptosis of CML stem/progenitor cells, we tested (in the presence of SCF, FL, and TPO) the impact of S63845, imatinib, and their combination on the ratio of activated caspase-3/7 expressing CD34+ stem/progenitor cells isolated from two CML patients at diagnosis, using fluorescence live cell microscopy (Fig. 7C, D; Supplementary Fig. 4). While each of the monotherapies moderately increased, combination of 1 μM imatinib and two- to low three-digit nanomolar concentrations of S63845 markedly enhanced further the rate of caspase-3/7 positive CML stem/progenitor cells in both samples.

Next, we analyzed (in the presence of SCF, FL and TPO) the effects of the drugs on the expression of apoptotic and pyroptotic markers and key antiapoptotic members of the BCL2 and BIRC gene families in CD34+ stem/progenitor cells isolated from a CML patient at diagnosis by western blot (Fig. 7E). S63845 monotherapy did not significantly affect BCL2, BCL-xL, MCL1, cIAP1, XIAP and survivin expression, while imatinib monotherapy moderately downregulated cIAP1 and survivin, but not the other antiapoptotic proteins. Contrary, combination treatment markedly downregulated the expression of MCL1, cIAP1 and XIAP, without affecting the expression of other antiapoptotic proteins. PARP cleavage could not be detected in cells left untreated or treated only with imatinib, while S63845 treatment minimally or moderately induced it in the absence or presence of imatinib, respectively. cIAP2, full length and cleaved GSDME protein, and the p30 GSDMD fragment could not be detected in cells left untreated or treated with any of the drugs or their combinations, while the production of the p43 GSDMD fragment was minimally or moderately induced by S63845 in the absence or presence of imatinib, respectively (data not shown).

These results and the observation that the CML stem/progenitor cells treated with the drug combination showed the characteristic morphology of apoptosis, but not pyroptosis (Supplementary Fig. 4), suggest that the combination treatment exerts its antiviability effect on these primary cells through the induction of apoptosis accompanied by the downregulation of MCL1 protein expression.

### Small-molecule MCL1 inhibition strongly enhances the anticlonogenic effect of imatinib on primary human CD34+ CML stem/progenitor cells

**Fig. 8:**
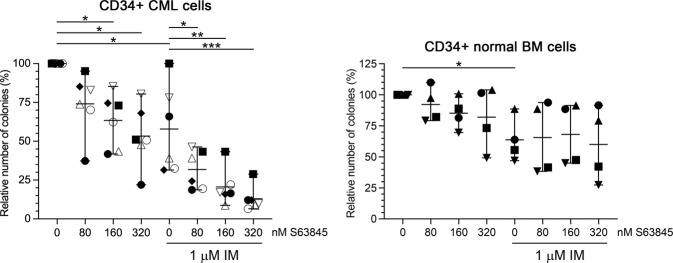
Effects of S63845 and imatinib administered as single drug or in combination on the colony forming capacity of primary human CD34+ stem/progenitor cells obtained from the peripheral blood of untreated CML patients in chronic phase or from the bone marrow of healthy donors. Relative colony forming capacity of CD34+ stem/progenitor cells obtained from the peripheral blood of untreated CML patients in chronic phase (left panel; *n* = 6 for all S63845 concentrations) or from the bone marrow (BM) of healthy donors (right panel; *n* = 4 for all S63845 concentrations) in the absence or presence of 1 μM imatinib (IM) and/or the indicated concentrations of S63845. Data represent mean with range derived from four (CD34+ normal BM cells) to six (CD34+ CML cells) independent experiments. *P < 0.05; **P < 0.01; ***P < 0.001. Patient no. 1: ⚫; patient no. 2: ◼; patient no. 5: ◆; patient no. 6: ○; patient no. 8: △; patient no. 9: ▽; healthy donor no. 1 ⚫; donor no. 2: ◼; donor no. 3: ▲; donor no. 4: ▼.

To assess the effect of the drugs on an additional potential surrogate marker for in vivo clinical drug response, we analyzed whether the treatments might also inhibit the colony forming capacity of CD34+ stem/progenitor cells isolated from six CML patients at diagnosis (Fig. 8 and Supplementary Fig. 3B, left panels). Similarly to the liquid cultures, 1 μM imatinib markedly reduced colony forming capacity of CML stem/progenitor cells in almost all the samples tested, while S63845 monotherapy exerted variable effects, with remarkable inhibition in some of the samples. More importantly, combination treatment with 1 μM imatinib and two- to low three-digit nanomolar S63845 exerted markedly stronger anticlonogenic effect than either of the monotherapies.

### Small-molecule MCL1 inhibition does not enhance the anticlonogenic effect of imatinib on primary human CD34+ normal bone marrow hematopoietic stem/progenitor cells

To test whether the observed anticlonogenic effect of S63845 on CD34+ CML cells is selective for the leukemic stem/progenitor cells, colony forming assays were also carried out on CD34+ stem/progenitor cells isolated from the bone marrow of four healthy donors (Fig. 8 and Supplementary Fig. 3B, right panels). In line with previous observations, S63845 monotherapy only minimally affected colony formation of normal hematopoietic stem/progenitor cells [10, 12]. In contrast, 1 μM imatinib as a single agent significantly suppressed their clonogenic capacity, a result well in line with previously published data [34]. More importantly, S63845 did not enhance this well-known inhibitory effect of imatinib on normal CD34+ hematopoietic stem/progenitor cells, suggesting a potential therapeutic window to efficiently target CML stem/progenitor cells without affecting the normal hematopoietic stem/progenitor cell population.

## Discussion

TKIs are highly effective in and have revolutionized CML therapy. Still, a wide majority of the patients consistently show molecular signs of remaining leukemic cells, probably at the stem/progenitor cell level, even after many years of continuous TKI treatment. As these cells appear capable of surviving TKI treatment, discontinuation of therapy is commonly followed by disease progression, as assessed by increasing BCR-ABL1 transcript levels. A critical mechanism behind the TKI resistance of these residual CML stem/progenitor cells is thought to be an enhanced activity of various BCR-ABL1-independent signaling pathways [1, 2]. Using low nanomolar concentrations of S63845, a novel highly specific and potent small-molecule MCL1 inhibitor, we could show for the first time that targeting MCL1 in the presence of BCR-ABL1 inhibition exerted a strong antitumorigenic effect on primary human CD34+ CML stem/progenitor cells. These results suggest that MCL1 might be one of the key survival factors that contribute to the BCR-ABL1-independent survival of CML stem/progenitor cells.

Previously, Kotschy et al. [10] showed that CML cells are relatively insensitive to S63845 treatment, possibly due to their high BCL-xL expression levels, which correlated with the resistance of hematological cancer cell lines to MCL1 inhibition. We could confirm the relative insensitivity of CML cells to S63845, but in contrast we also showed that TKI treatment induced rapid sensitization of CML cells to the antitumorigenic effects of this drug. Furthermore, the rapid and strong activation of caspase-3/7, induction of PARP cleavage, and that cell death could be blocked by zVAD, a pan-caspase inhibitor proved that the drug combination exerted its antitumorigenic effect mainly through the induction of apoptosis both in CML lines and primary CML stem/progenitor cells, although variable proportions of cells in the CML lines also showed characteristics of GSDME-dependent pyroptosis. In line with these results, combination treatment but none of the monotherapies markedly downregulated MCL1 protein expression in K562 and CML stem/progenitor cells. On the contrary, MCL1 protein expression did not decrease upon any of the treatments in TCCS cells, suggesting that downregulation of MCL1 protein expression is not essential for the inhibitory effect of S63845. Furthermore, western blot analysis showed that BCL-xL expression was not significantly downregulated by any of the treatments in K562 and cytokine-treated CML stem/progenitor cells, suggesting that other mechanisms might be responsible for the TKI-induced shift in S63845 sensitivity of CML cells.

We could show that a combination of S63845 either with ATP-competitive TKIs or with asciminib, a highly selective, allosteric ABL1 kinase inhibitor [24] exerted similar synergistic proapoptotic effect on CML cells. This suggests that the synergistic effects of the drug combinations are independent of the potential off-target effects of the TKIs [25, 26]. Indeed, using T315I mutation harboring sublines of well-characterized CML lines we proved that inhibition of BCR-ABL1 kinase activity is essential, while the off-target effects of TKIs do not play a key role in the synergistic proapoptotic interaction between MCL1 inhibition and TKI therapy in CML cells.

A key issue when targeting MCL1, especially in combination treatments, is the tolerability of such a drug (combinations) in humans, as conditional gene knockout studies revealed an essential role for MCL1 in the survival of several critical cell types, including hematopoietic stem cells and cardiomyocytes [6]. However, several lines of evidence suggest that irreversible loss of the whole MCL1 protein due to gene knockout might exert a completely different effect than intermittent periods of pharmacological inhibition of a specific function of MCL1. For example, S63845 monotherapy in efficacious concentrations did not show any toxic effects on normal hematopoietic stem/progenitor cells [10, 12]. More importantly, we showed that combination treatment with antitumorigenic concentrations of S63845 and imatinib does not significantly potentiate the anticlonogenic effect of imatinib on normal human hematopoietic stem/progenitor cells, suggesting that a suitable therapeutic window for combination treatment with a small-molecule MCL1 inhibitor and a TKI might be achievable in CML patients. Although more extensive tolerability data are still pending, this assumption is also supported by several successfully completed phase I/II clinical trials in various types of cancers with AT-101, a small molecule simultaneously targeting MCL1, BCL2 and BCL-xL [7].

MCL1 expression can be upregulated in CML cells either by BCR-ABL1 signaling [9] or potentially by the microenvironment, mainly through signaling of the cytokine receptors which play an important role in the TKI resistance of CD34+ CML stem/progenitor cells [35, 36]. These cytokine receptor signals also prevent normal hematopoietic stem/progenitor cells from spontaneous apoptosis, at least partially through the upregulation of MCL1 [37]. In line with these observations, we noted that the combination of MCL1 inhibition and TKI therapy exerted strong synergistic antitumorigenic effects on CML stem/progenitor cells, both in the absence or presence of these cytokines. However, the same drug combination in the presence of these cytokines did not significantly reduce colony formation of normal hematopoietic stem/progenitor cells. These results suggest that while cytokine induced MCL1 upregulation may be essential for the survival of CML stem/progenitor cells, it plays only a marginal role in the survival of normal hematopoietic stem/progenitor cells.

In summary, our data demonstrate a consistent pattern where addition of an MCL1 inhibitor to imatinib strongly and synergistically enhanced the antitumorigenic impact of the TKI on primary human CD34+ CML stem/progenitor cells and on CML cell lines, while the effects of MCL1 inhibitor monotherapy were less conclusive. Importantly, MCL1 inhibition did not enhance the anticlonogenic effect of imatinib on normal hematopoietic stem/progenitor cells, suggesting a therapeutic window for an MCL1 inhibitor and TKI combination treatment specifically targeting CML cells. Recent recommendations highlight treatment-free remission (TFR) as a new significant goal of CML management, with sustained deep molecular response (DMR; BCR-ABL1 < 0.01% IS) as a prerequisite for a TKI discontinuation attempt [38]. Thus, for CML patients not reaching DMR on prolonged TKI monotherapy and for whom achieving TFR is considered important, or for those not even reaching major molecular response (BCR-ABL1 < 0.1% IS), the concept of adding a non ABL-directed, molecularly targeted drug to a TKI may appear highly appropriate. In agreement, several new compounds are already being explored in this setting in ongoing clinical trials [2, 5]. Our data suggest that small-molecule MCL1 inhibitors can be next in line.

## Supplementary information

Supplemental Figure Legends

Supplemental Table 1

Supplemental Figure 1

Supplemental Figure 2

Supplemental Figure 3

Supplemental Figure 4
