# Supplementary material for: Combination of tyrosine kinase inhibitors and the MCL1 inhibitor S63845 exerts synergistic antitumorigenic effects on CML cells
Source: Cell Death Dis. 2021 Sep 25;12(10):875. doi: 10.1038/s41419-021-04154-0 (PMC8464601; doi:10.1038/s41419-021-04154-0)
Supplement: Supplementary file 1 — Supplemental Figure Legends [file 41419_2021_4154_MOESM1_ESM.docx]

**Supplemental Figure legends**

**Supplemental Figure 1. Direct sequencing of the genomic region of the ABL1 gene harboring codon 315 in the parental K562 cells and its imatinib resistant sublines generated by HR using the CRISPR/Cas9 system.** A 427 bp fragment of the ABL1 gene harboring codon 315 at exon 6 was amplified by PCR from the genomic DNA of the parental and imatinib resistant sublines of K562, followed by the direct sequencing of the PCR products. Wild-type and mutated amino acid and genomic DNA sequences are indicated at the top of the panel. Arrowheads indicate mutations generated by HR.

**Supplemental Figure 2. Combination treatment with S63845 and imatinib synergistically induces caspase-3/7 activation in KCL-22 cells.** Proportion of active caspase-3/7 positive KCL-22 cells left untreated or treated for 68 hours with 1 μM imatinib and/or the indicated concentrations of S63845, analyzed by fluorescence live cell microscopy. Data represent mean with standard deviation derived from three technical replicates. Experiments were performed twice with similar results.

**Supplemental Figure 3.** Absolute numbers of viable cells (A) and colonies (B) obtained in the experiments presented in Figures 7A and 8, respectively.

**Supplemental Figure 4.** Representative fluorescence live cell microscopy images obtained in the experiment presented in Figure 7C.
