# Supplementary material for: Combination of tyrosine kinase inhibitors and the MCL1 inhibitor S63845 exerts synergistic antitumorigenic effects on CML cells
Source: Cell Death Dis. 2021 Sep 25;12(10):875. doi: 10.1038/s41419-021-04154-0 (PMC8464601; doi:10.1038/s41419-021-04154-0)
Supplement: Supplementary file 2 — Supplemental Table 1 [file 41419_2021_4154_MOESM2_ESM.docx]

**Supplemental Table 1. List of antibodies used for western blotting**

rabbit anti-Bcl-2 (clone D55G8; CST; Cat. No.: 4223)

rabbit anti-Bcl-xL (clone 54H6; CST; Cat. No.: 2764)

rabbit anti-cIAP1 (clone D5G9; CST; Cat. No.: 7065)

rabbit anti-cIAP2 (clone 58C7; CST; Cat. No.: 3130)

rabbit anti-Caspase-3 (clone D3R6Y; CST; Cat. No.: 14220)

rabbit anti-Caspase-7 (clone D2Q3L; CST; Cat. No.: 12827)

rabbit anti-DFNA5/GSDME-N-terminal (clone EPR19859; Abcam; Cat. No.: ab215191)

rabbit anti-Gasdermin D (clone E8G3F; CST; Cat. No.: 97558)

rabbit anti-Mcl-1 (clone D35A5; CST; Cat. No.: 5453)

rabbit anti-PARP (CST; Cat. No.: 9542)

rabbit anti-XIAP (clone 3B6; CST; Cat. No.: 2045)

rabbit anti-Survivin (clone 71G4B7; CST; Cat. No.: 2808)

rabbit anti-β-Actin (clone 13E5; CST; Cat. No.: 5125)

CST, Cell Signaling Technology
