# Supplementary figures and images for: Combination of tyrosine kinase inhibitors and the MCL1 inhibitor S63845 exerts synergistic antitumorigenic effects on CML cells
Source: Cell Death Dis. 2021 Sep 25;12(10):875. doi: 10.1038/s41419-021-04154-0 (PMC8464601; doi:10.1038/s41419-021-04154-0)

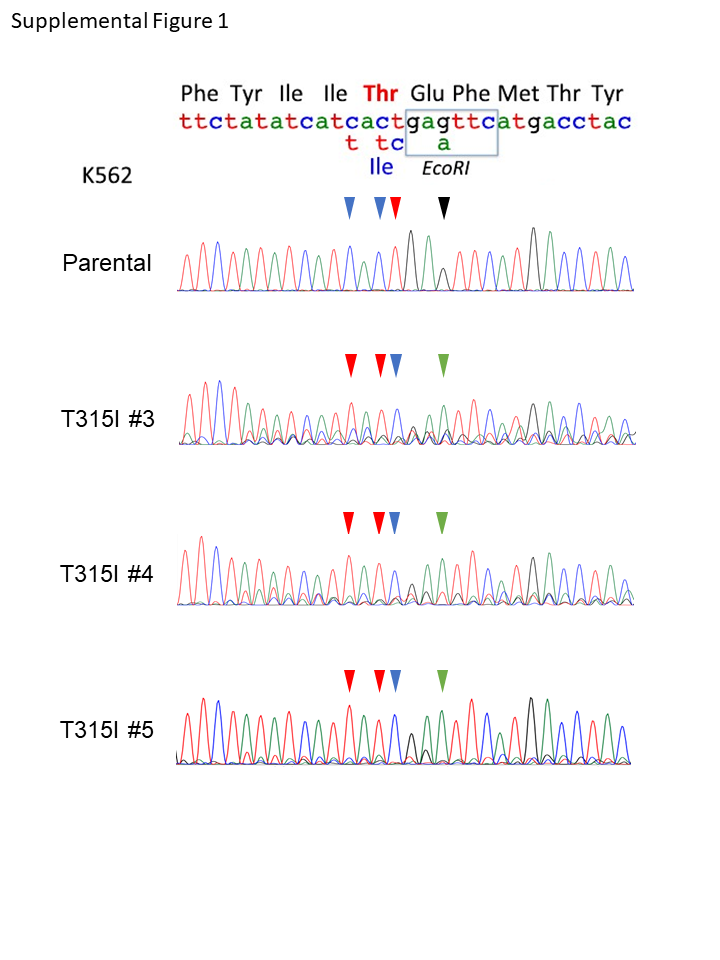

Supplement: Supplementary file 3 — Supplemental Figure 1 [file 41419_2021_4154_MOESM3_ESM.tif]

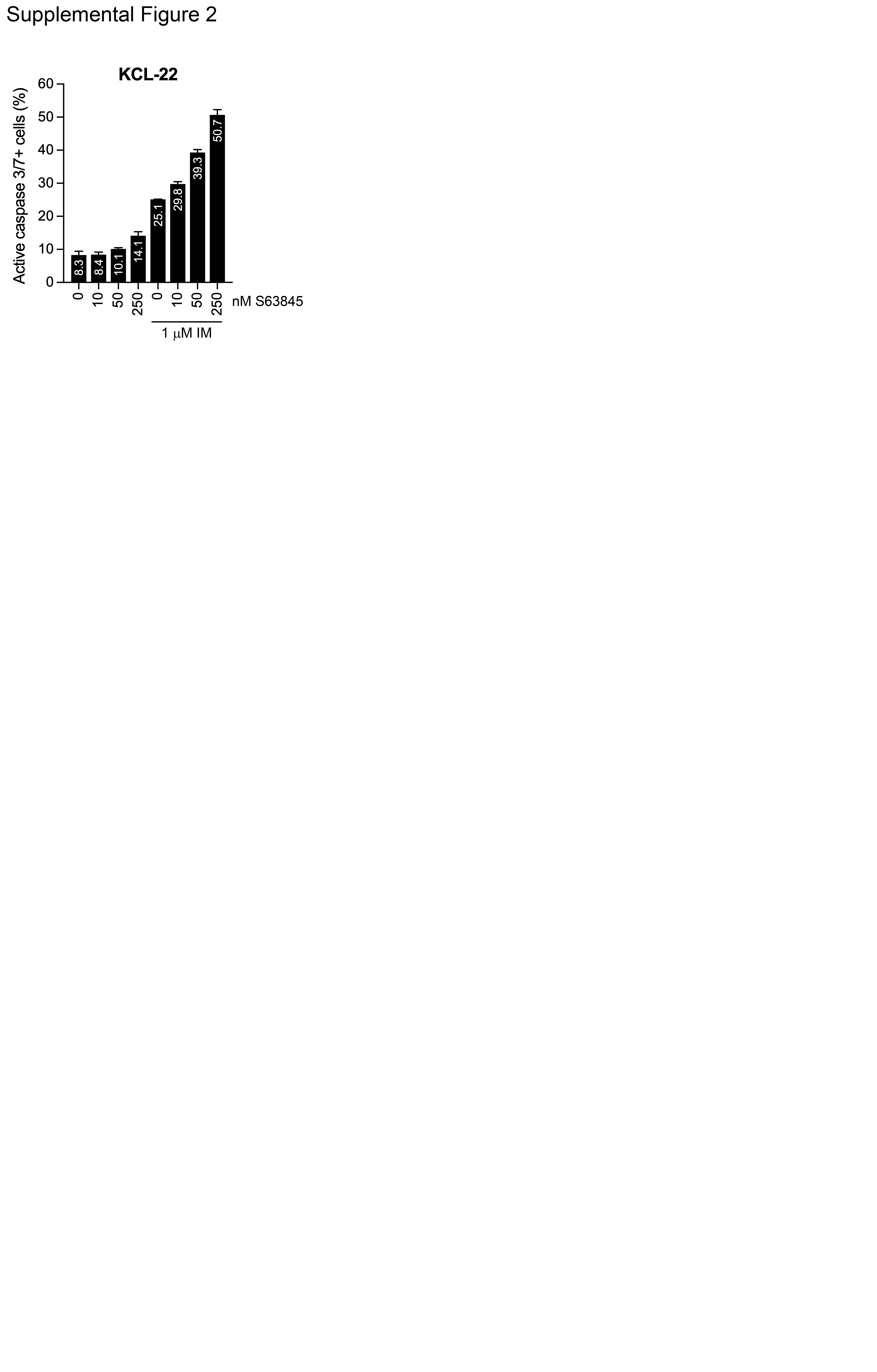

Supplement: Supplementary file 4 — Supplemental Figure 2 [file 41419_2021_4154_MOESM4_ESM.tif]

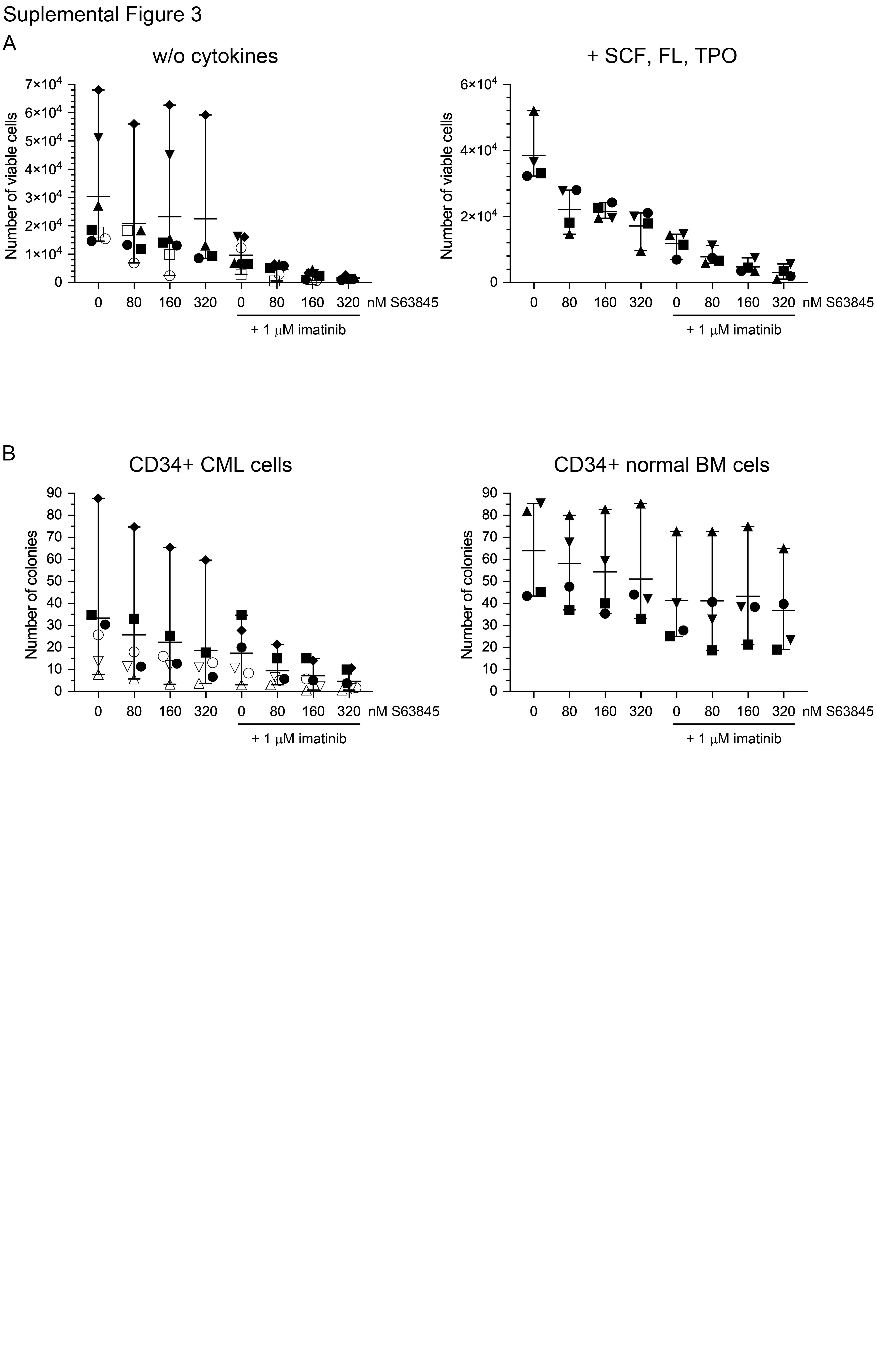

Supplement: Supplementary file 5 — Supplemental Figure 3 [file 41419_2021_4154_MOESM5_ESM.tif]
